# Suicide risk following ED presentation with self-harm varies by hospital

**DOI:** 10.1007/s00127-023-02561-6

**Published:** 2023-10-20

**Authors:** Siobhan Murphy, Emma Ross, Denise O’Hagan, Aideen Maguire, Dermot O’Reilly

**Affiliations:** 1 Centre for Public Health, Institute of Clinical Science, Queen’s University Belfast, Royal Victoria Hospital, Belfast, BT12 6BJ Northern Ireland; 2 Public Health Agency, Belfast, Northern Ireland

**Keywords:** Self-harm, Mortality risk, Suicide, ED

## Abstract

**Background:**

Patients presenting to Emergency Department (ED) with self-harm are recognized to be at high risk of suicide and other causes of death in the immediate period following ED presentation. It is also recognized that there is a large variation in the management and care pathways that these patients experience at EDs.

**Aims:**

This study asks if there is a significant variation in mortality risk according to hospital attended and if this is explained by differences in care management.

**Methods:**

Population-wide data from the Northern Ireland Registry of Self-Harm from April 2012 were linked with centrally held mortality records to December 2019, providing data on self-harm type and ED care. Cox proportional hazards models analyzed mortality risk, coded as suicide, all-external causes and all-cause mortality.

**Results:**

Analysis of the 64,350 ED presentations for self-harm by 30,011 individuals confirmed a marked variation across EDs in proportion of patients receiving mental health assessment and likelihood of admission to general and psychiatric wards. There was a significant variation in suicide risk following attendance according to ED attended with the three-fold range between the lowest (HR_adj_ 0.32 95% CIs 0.16, 0.67) and highest. These differences persisted even after adjustment for patient characteristics, variation in types of self-harm, and care management at the ED.

**Conclusions:**

This study suggests that while the management of self-harm cases in the ED is important, it is the availability and access to, and level of engagement with, the subsequent management and care in the community rather than the immediate care at EDs that is most critical for patients presenting to ED with self-harm. However, the initial care in ED is an important gateway in initiating referrals to these services.

## Introduction

Self-harm (SH) is commonly a manifestation of poor mental health and makes up a significant proportion of the workload of emergency departments (ED) [1, 2]. There is evidence that the prevalence of ED attendances with SH is increasing [3], and although many people who self-harm do not present to hospital EDs [4], those that do so are recognized to be at very high risk of suicide, fatal alcohol or drug poisoning, and other causes of premature mortality [5–7]. That this excess risk is particularly high in the month following ED attendance [8], underscores the importance of how EDs manage the complex physical and psychological needs of patients presenting with SH [9].

The UK, like other countries, has introduced national guidelines for the assessment and management of those presenting with SH [10, 11]. The most recent draft guidelines [12], inter alia recommends that all people presenting to an ED with SH are offered referral to liaison psychiatry services (or an equivalent specialist mental health service) as soon as possible after arrival, for a psychosocial assessment and support alongside their medical care. This should be offered to all patients without recourse to risk stratification tools as these cannot accurately predict risk of future self-harm or suicide. [12]

In a recent paired review, House & Owen concluded that there was little evidence of progress in the last 25 years [13], while Kapur, although more sanguine, acknowledged that the implementation gap for self-harm services remains substantial [9]. Despite the introduction of national guidelines, recent evidence demonstrates there continues to be significant variation in the levels and type of care offered by hospitals, with disquietingly contrasting patient experiences of care provided and received [14], and calls for improvements in levels of care [15]. One study showed a 2.5-fold variation in admission rates between hospitals in London [16], and Walker et al. in a recent survey of all hospitals in England found while the majority provided 7-day-based liaison psychiatry services, less than half provided 24-h coverage [17]. Many studies show a significant proportion of patients leave without being seen or assessed [18]. Cooper et al. found little reduction in variability between hospitals in England after the introduction of national guidelines [19]. Studies in the Republic of Ireland, based on national registry data, have found similar levels of variation in the adherence to recommended care pathways following presentation to ED [20], and that while individual factors are important, hospital-level elements such as the availability of onsite mental health staff, explained almost all the variation in the proportion of patients receiving a psychosocial assessment [21].

In most countries, the only nationwide, routinely available data on service use for self-harm are based on hospital admissions. However, in Northern Ireland and the Republic of Ireland there are established nationwide self-harm registers that capture comprehensive data on self-harm presentations to EDs [22, 23] regardless of whether or not the patient was admitted to hospital. Access to this type of data at a national level provides information on specific service capacity and how this may vary and the effects this may have on mortality risk. The aim of the current study is to determine if the risk of death following ED presentation with SH differs according to hospital attended, and if these differences are associated with the variation in care provided at those hospitals. Although the primary focus of the paper related to suicide, deaths from all-external causes and all-cause mortality are included for comparison.

## Methods

This is a population-wide record linkage-based cohort study of patients presenting with SH to EDs in Northern Ireland (NI). Data on SH come from the Northern Ireland Registry of Self-Harm (NIRSH) which, since April 2012 has employed trained data collectors using standardised criteria to extract data on all episodes of intentional self-harm regardless of suicidal intent or motivation presenting to all eleven EDs in NI. Episodes of self-harm carried out by individuals with a learning disability were excluded, as self-harm in this population differs qualitatively to those in the general population.

The NIRSH records data related to the individual, such as age at presentation, sex, and usual residential address for each of the eleven hospitals attended. The methods of self-harm are coded according to International Classification of Disease, Tenth Revision (ICD-10) codes (X60-X84). The main cause of self-harm is grouped here as drug overdose/self-poisoning (Overdose; ICD 10 X60-X69); self-cutting (Cutting; ICD 10 X78); or attempted hanging/drowning/other methods and mixed methods of self-harm (Hanging/drowning; ICD10 X70- X84). Assessment by a mental health professional while in the ED or referred for later mental health assessment in the community/ward was also recorded as was ED management and next care (e.g. discharged following treatment (discharged); left before assessment/management decision or refused admission (labelled as ‘refused’ in this paper), or admission to a general or psychiatric ward). The patient’s unique Health and Care Number (HCN), which is used throughout the health service in Northern Ireland, is also captured. This enabled the identification of repeat attenders irrespective of ED of presentation. Episode details are recorded on password protected data forms before encryption and entry into a data entry system for analysis. NIRSH is subject to routine audits and quality checks.

Data for this study were extracted for all SH presentations registered between 1st April 2012 and 31st December 2019 and linked to the Health Service Central Registration Spine using the HCN as linkage key. This spine receives quarterly updates on date and cause of death from the General Register’s Office, though there are known delays with suspected deaths by suicide that are processed through a coroner’s court. Deaths by suicide were defined as ICD-10 codes indicating suicide (intentional self-harm ICD10: X60-X84) and sequalae of intentional self-harm (ICD10: Y87.0). External deaths were defined as those coded as V01-Y98. The patient’s address on the population spine was used to append information about the characteristics of their area of residence. Urban/rural habitation was based on a classification of settlements [24] and grouped into three approximately equal groups; urban (comprising the two largest cities), intermediate, or rural (settlements with less than 2250 people). Area deprivation was based on the Northern Ireland Multiple Deprivation Measure [25] and divided into quintiles ranging from least deprived to most deprived.

The project was designed in collaboration with the organisation that hosts the Registry and approved by Honest Broker Service Governance Board. Ethical approval was granted by the Research Ethics Committee (REC)—REF 19/LO/1601. All data were linked by the unique identifier and then replaced with a de-identified dataset that was made available to the research team through the Business Services Organisation’s online Secure Research Platform. The final anonymous research dataset was made available to the named research group on network-isolated computers within the Trusted Research Environment. All statistical outputs were subject to additional disclosure control measures, including restrictions on cell numbers to protect confidentiality. Individual hospital identifiers were additionally replaced with anonymous markers.

### Statistical analysis

The analysis proceeded in three stages: the first used descriptive statistics, to portray the variations in socio-demographic characteristics of patients presenting to these eleven hospitals and type of self-harm for each of these presentations. The next stage used logistic regression to quantify the variation across hospitals in likelihood of receiving a specialist mental health assessment while in the ED or referral specialist mental health assessment, and finally multinomial regression to examine the variations in next care (left or refused care, or admission to general or psychiatric hospital) with ‘discharged’ as the reference category. In both these stages of analyses we used episode level presentations to EDs.

The significance of adding hospital dummies to the survival models was tested using the likelihood ratio test for hospital term in the models. This is considered a more powerful approach and circumvents the findings of spurious variations arising from choice of reference hospital. Separate Cox proportional hazards regression models were then used to examine the variation in risk of death from suicide across hospitals, death due to external causes, and all-cause mortality. Individuals were followed up from the date of their index presentation until either death or the end of the follow-up period December 2019 (whichever came first). Models examining cause-specific mortality were censored at date of death (right censoring) for any causes of death to account for competing risks.

Further models examined variation in survival by ED, with stepwise adjustment for patient characteristics, means of SH, and finally whether they had received a mental health assessment/referral, and their management and next care, to determine the extent these factors explained any variation in mortality risk according to hospital attended. To account for repeat attendances, we adjusted for the number of attendances in the final models.

A sensitivity analyses repeated the examination of mortality variation across hospitals to determine if the patterns were consistent across early (2012–2015) and later (2016–2019) time periods as it is recognised that care and management practices have changed over this period. All analyses were conducted using STATA 15.

### Reporting

We produced a detailed analysis protocol prior to undertaking the analysis. We followed the Strengthening the Reporting of Observational studies in Epidemiology (STROBE) and Reporting of studies Conducted using Observational Routinely collected Data (RECORD) checklists to guide transparent reporting of this cohort study.

## Results

### Self-harm presentations

There were 64,350 ED presentations (episodes) of self-harm during April 2012 to December 2019 made by 30,011 individuals. Table 1 confirms the usual socio-economic and demographic patterns of people in NI presenting to ED with self-harm. Females accounted for a slightly higher proportion of all self-harm episodes than males (51.0% vs. 49.0%), and presentations were more common in those aged 20–29 years and lowest in those 50 years-or-over (29.0% vs. 16.0%). There was a disproportionate number from the more disadvantaged areas. Overdose was the most common method of self-harm (62.0%), followed by self-cutting (20.0%), and more lethal methods of self-harm such as hanging and drowning or a combination of methods (18.0%) were the least common. Approximately two-thirds (69.2%) of SH episodes were repeat occurrences within the study period. Table 1 also shows that there was little significant variation in the demographic characteristics of self-harm presenters across EDs, but that the marked deprivation and urban/rural variations reflected the characteristics of the corresponding hospital catchment areas. There was also some modest variation in the type of self-harm; the most notable, that Hospital B experienced a significantly larger number of attempted “drowning” episodes than any of the other hospitals.

**Table 1:** Variations in socio-demographic characteristics and type of self-harm for patients presenting at different hospital Emergency Departments from April 2012 to December 2019

	Anonymous hospital code											
	A	B	C	D	E	F	G	H	I	J	K	Total
Presentation (*n*)	10,563	8891	3375	7886	7586	8142	3209	8822	3831	1279	766	64,350
Age (years)												
10–19	21.1	17.3	17.9	15.5	25.0	16.3	17.2	18.6	16.3	16.5	19.6	18.6
20–29	29.8	29.4	27.7	32.4	26.6	31.1	24.5	27.5	28.7	33.0	22.1	29.1
30–39	18.5	26.0	17.8	21.0	16.1	18.8	18.0	17.7	16.9	16.9	18.8	19.3
40–49	15.1	14.8	18.0	15.7	16.5	17.9	21.0	19.5	18.4	17.1	19.3	17.0
50 +	15.5	12.6	18.5	15.5	15.8	15.9	19.3	16.8	19.7	16.5	20.2	16.0
Sex												
Male	50.1	47.5	46.0	54.5	43.8	51.4	46.4	48.8	49.1	48.6	46.5	49.0
Female	49.9	52.6	54.0	45.5	56.3	48.7	53.6	51.2	50.9	51.4	53.5	51.0
Single person household												
No	83.7	84.9	75.3	80.3	83.4	83.6	83.3	83.7	83.4	80.8	84.2	82.9
Yes	12.4	12.3	14.6	14.7	14.0	12.4	13.1	14.0	13.3	16.1	12.0	13.3
Missing	3.9	2.8	10.2	5.0	2.7	4.0	3.6	2.4	3.3	3.1	3.8	3.8
Urban/rural												
Urban	57.1	57.6	1.9	68.4	20.9	3.3	1.6	5.1	4.3	14.3	3.9	30.1
Intermediate	34.4	16.9	46.7	26.4	61.5	65.6	60.6	71.6	63.1	67.9	52.1	47.8
Rural	6.9	22.6	48.7	4.0	16.5	29.1	36.5	21.9	31.6	16.6	43.2	20.5
Missing	1.6	2.9	2.7	1.2	1.2	2.0	1.3	1.4	1.0	1.3	0.8	1.7
Area deprivation												
Affluent	14.7	4.4	1.1	10.0	15.8	10.7	1.9	19.0	4.4	15.3	3.1	10.8
Second	19.0	4.1	8.3	14.5	25.8	14.0	6.3	22.6	8.5	29.0	24.4	15.5
Third	19.4	14.3	29.6	15.0	20.0	22.5	18.5	21.5	18.7	23.0	20.0	19.4
Fourth	20.3	16.4	23.9	19.6	20.8	24.1	30.1	20.4	27.0	8.5	35.9	21.3
Deprived	25.0	57.8	34.3	39.8	16.5	26.8	41.8	15.1	40.4	22.9	15.8	31.3
Missing	1.6	2.9	2.7	1.2	1.2	2.0	1.3	1.4	1.0	1.3	0.8	1.7
Number of presentations												
One	29.9	26.6	32.4	27.0	33.2	31.3	36.2	33.2	32.2	32.3	35.6	30.8
Two or more	70.1	73.4	67.6	73.0	66.8	68.8	63.8	66.8	67.8	67.7	64.4	69.2
Type of self-harm												
Self-poisoning	66.6	54.3	64.6	59.0	63.1	60.5	66.2	65.6	63.3	62.7	59.1	62.2
Cutting	24.8	18.3	17.5	24.2	21.8	22.5	15.5	17.9	18.0	22.4	24.9	20.0
Hanging/drowning/other	8.6	27.4	17.8	16.9	15.1	17.0	18.3	16.5	18.7	14.9	15.9	17.8

Data represent the percentage of presentations at each ED

### Care and management

Table 2 and 3 shows the variations in mental health assessment/referral and next care modalities for episodes of SH. Over the 8-year period an average of 66% of people presenting at ED are documented in ED notes as having received or being referred for a mental health assessment, though it should be noted that this proportion has increased to over 80% in recent years (results not shown here). While the majority of attendances at each site received an assessment or referral, there was a two-fold range in the odds of this, with most hospitals, with the exception of Hospital J, significantly higher than Hospital A (Table 3). The largest proportion of attendances (46.7%) was admitted to general medical or surgical or short stay wards, a further 39.2% were discharged directly from the ED following treatment, and 5% were admitted to a psychiatric ward; approximately 9.2% refused treatment or left before being seen by an ED clinician (Table 2). Hospital D had the higher proportion (15.2%) of people leaving before being seen by an ED clinician or refusing treatment, compared to only 3.3% for Hospital E. The largest variation (ten-fold) was in the proportions admitted to a psychiatric hospital (range 1.2% for Hospital A: 12.3% for Hospital C), and this difference persisted, though a little attenuated, even after adjustment for characteristics of the patients and the types of self-harm presenting (Table 3).

**Table 2:** Proportion of patients presenting with self-harm receiving a mental health assessment and variation in the type of next care provided according to hospital attended between 2012 and 2019

Hospital	Received mental health assessment/referral	Next care			
		Discharged	Refused treatment	Admitted to general ward	Admitted to psychiatric ward
A	54.1	39.0	11.4	48.4	1.2
B	65.1	37.1	13.3	42.5	7.2
C	64.4	42.8	7.0	37.9	12.3
D	62.0	40.6	15.2	42.1	2.0
E	76.3	49.2	3.3	42.0	5.6
F	71.4	35.0	8.2	48.4	8.4
G	66.5	35.1	6.9	52.8	5.2
H	74.0	28.4	4.9	64.0	2.8
I	67.1	55.4	10.3	30.7	4.7
J	55.0	40.8	7.2	45.7	6.3
K	75.1	42.6	5.2	40.9	11.4
**All hospitals**	**66.3**	**39.2**	**9.2**	**46.7**	**5.0**

Note: logistic regression models adjusted for age, sex, single person households, area deprivation, urban/rural residence, and method of self-harm. The multinomial regression also adjusts for number of self-harm presentations, and whether the patient received a mental health assessment

**Table 3:** Likelihood of receiving mental health assessment and Relative Risk Ratios of refusing care, or being admitted to a general or psychiatric hospital, compared to standard according to hospital attended. Data are, respectively, Odds Ratios and Relative Risks from fully adjusted* logistic regression and multinomial regression analyses

Hospital	Received mental health assessment/referral	Next care (relative to discharge)		
		Refused treatment	Admitted to general ward	Admitted to psychiatric ward
A	1.00	1.00	1.00	1.00
B	1.50 (1.41, 1.60)	1.48 (1.33, 1.64)	1.09 (1.01, 1.16)	4.95 (4.04, 6.07)
C	1.16 (1.06, 1.27)	0.64 (0.54, 0.75)	0.69 (0.62, 0.76)	7.38 (5.91, 9.21)
D	1.56 (1.47, 1.67)	1.37 (1.24, 1.51)	0.92 (0.86, 0.99)	1.47 (1.16, 1.88)
E	2.21 (2.06, 2.37)	0.32 (0.28, 0.38)	0.81 (0.76, 0.87)	3.19 (2.58, 3.93)
F	1.91 (1.78, 2.04)	1.10 (0.97, 1.24)	1.34 (1.24, 1.44)	5.91 (4.81, 7.27)
G	1.55 (1.41, 1.70)	0.85 (0.72, 1.01)	1.27 (1.15, 1.40)	3.53 (2.74, 4.55)
H	2.43 (2.27, 2.60)	0.91 (0.80, 1.04)	2.26 (2.10, 2.43)	2.43 (1.93, 3.06)
I	1.45 (1.33, 1.59)	0.72 (0.62, 0.83)	0.43 (0.39, 0.47)	2.09 (1.64, 2.67)
J	0.92 (0.81, 1.05)	0.54 (0.42, 0.69)	0.86 (0.75, 0.99)	4.50 (3.33, 6.09)
K	2.08 (1.73, 2.48)	0.57 (0.40, 0.80)	0.89 (0.74, 1.05)	7.07 (5.20, 9.61)

Note: logistic regression models adjusted for age, sex, single person households, area deprivation, urban/rural residence, and method of self-harm. The multinomial regression also adjusts for number of self-harm presentations, and whether the patient received a mental health assessment

### Risk of death according to ED attended

Almost 6% (*n* = 1739) of individuals who had presented with an index episode of self-harm between April 2012 and December 2019 died during the follow-up period up to December 2019. Of these deaths, 47.8% (*n* = 831) were due to external causes. Suicide is a sub-set of external causes and accounted for 19.4% (*n* = 337) of the total deaths.

The fully adjusted models from the survival analysis confirmed the findings of earlier studies [4–10] in that suicide risk was three-times higher for those presenting with drowning or hanging or mixed methods, twice as high for males as for females and increased modestly with age but with no association to household composition or area characteristics (full models available on request). Results also show that risk was lower (albeit not significant lower) in those who received a specialist mental health assessment while in the ED (HR_adj_ 0.76 95% CI 0.56, 1.05) and significantly lower when a referral was made for later mental health assessment (HR_adj_ 0.69 95% CI 0.52, 0.91), but was higher in those admitted to a general or a psychiatric ward (HR_adj_ 1.43 95% CI 1.07, 1.90 and HR_adj_ 2.11 95% CI 1.34, 3.33 respectively, compared to those discharged from the ED after treatment).

Survival analysis revealed a difference between hospitals for death due to suicide as an outcome (likelihood ratio test for hospital term (*p* = 0.0004), but not for deaths due to all external causes of death (*p* = 0.128) or for all-cause mortality risk (*p* = 0.330). Table 4 shows a three-fold range in survival for deaths by suicide across EDs, with the highest mortality risk at Hospital A and the lowest at Hospital G (HR_adj_ 0.32 95% CI 0.16, 0.67). This range of difference persisted even after adjustment for variations in the types of people presenting, the type of self-harm, and the variation in management they received at the ED including whether or not they were referred for psychosocial assessment or admitted to hospital. It has been suggested that self-harm patients who leave the ED without a specialist mental health assessment may be at high risk of suicide but the differences across EDs were unaffected by excluding those who refused care or left without being seen (results available on request). The almost 50% lower risk of suicide for patients attending Hospital B, compared to Hospital A (HR 0.53 95%CI 0.34, 0.81), also remained in models that excluded patients presenting with attempted drowning/hanging, which were disproportionately represented at Hospital B (results available on request). Collectively the three hospitals with the lowest risk of suicide accounted for 43.2% of all SH presentations (Table 1). Further sensitivity analysis showed very similar orders of variations for suicide risk across EDs (though with wider confidence intervals) when the analysis was restricted to people presenting over the last four years. There was no significant variation in all-cause mortality risk between hospitals with no hospital significantly different from Hospital A in the fully adjusted models. The variation in risk of death from es across sites was more muted than for suicides with two hospitals (B and E) significantly lower than Hospital A.

**Table 4:** Variation in mortality from suicide, all external causes and all-causes after presentation with SH according to hospital attended. Data represent hazard ratios and 95%confidence intervals for the unadjusted and fully adjusted models

	Suicides (*n* = 337)		External causes (*n* = 831)		All causes (*n* = 1739)	
	Unadjusted	Adjusted	Unadjusted	Adjusted	Unadjusted	Adjusted
A	1.00	1.00	1.00	1.00	1.00	1.00
B	**0.57 (0.38, 0.87)**	**0.53 (0.34, 0.81)**	**0.76 (0.59, 0.99)**	**0.75 (0.57, 0.98)**	0.90 (0.75, 1.08)	0.89 (0.74, 1.07)
C	1.04 (0.67, 1.63)	0.90 (0.54, 1.48)	0.95 (0.70, 1.31)	0.99 (0.70, 1.41)	0.97 (0.77, 1.21)	1.00 (0.78, 1.28)
D	0.75 (0.51, 1.10)	0.76 (0.51, 1.14)	0.98 (0.77, 1.25)	0.96 (0.75, 1.24)	0.95 (0.79, 1.13)	0.92 (0.76, 1.10)
E	**0.39 (0.25, 0.63)**	**0.39 (0.24, 0.63)**	**0.65 (0.50, 0.84)**	**0.73 (0.55, 0.96)**	**0.77 (0.64, 0.92)**	0.83 (0.68, 1.01)
F	**0.71 (0.48, 1.03)**	**0.62 (0.40, 0.96)**	0.84 (0.66, 1.08)	0.86 (0.65, 1.14)	0.96 (0.81, 1.14)	1.00 (0.83, 1.22)
G	**0.37 (0.18, 0.73)**	**0.32 (0.16, 0.67)**	**0.67 (0.46, 0.96)**	0.71 (0.48, 1.06)	0.84 (0.66, 1.07)	0.82 (0.63, 1.07)
H	0.87 (0.61, 1.23)	0.77 (0.52, 1.14)	0.89 (0.70, 1.12)	0.92 (0.71, 1.20)	0.98 (0.83, 1.16)	0.98 (0.81, 1.18)
I	0.70 (0.43, 1.14)	0.67 (0.39, 1.14)	**0.65 (0.46, 0.91)**	0.70 (0.48, 1.02)	0.84 (0.67, 1.06)	0.89 (0.70, 1.14)
J	1.10 (0.58, 2.07)	0.93 (0.48, 1.78)	0.95 (0.60, 1.05)	0.91 (0.57, 1.45)	1.08 (0.79, 1.48)	1.04 (0.75, 1.43)
K	0.83 (0.36, 1.90)	0.68 (0.29, 1.60)	0.93 (0.55, 1.58)	0.94 (0.54, 1.63)	1.22 (0.87, 1.70)	1.20 (0.85, 1.71)

Adjusted for age, sex, single person households, area deprivation, urban/rural residence, method of self-harm, number of self-harm presentations, whether received mental health assessment and whether discharged, refused care or admitted to general/psychiatric hospital

## Discussion

The study confirmed the established socio-demographic patterns and area-based variations of those presenting to EDs with self-harm, along with their marked increased risk of death from suicide and other external causes of death consistent with other studies [4–8]. It also supports previous studies demonstrating large variation in the management of patients who present to EDs with SH, including the proportions receiving mental health assessment or referral, and the proportions admitted to general or psychiatric hospitals. [17–20] Results show for the first time a significant three-fold variation across hospitals in suicide risk for patients attending EDs with SH. This mortality difference persisted even after adjustment for variations in the type of patients attending the different hospitals, the variation in methods of self-harm and the variations in the provisions of care and management experiences of these patients at ED.

There is a range of possible explanations for the mortality differences according to ED attended that requires some consideration. The first, that this represents an artefact arising from differences in clinical record keeping, cannot be entirely discounted. Artefacts due to the data collection process should be minimal given the quality assurance measures that include audits and cross-validation checks of extraction and coding at individual sites. It is also possible that the mortality differences are due to unmeasured differences in the types of people presenting to EDs, though we note that there was little attenuation in variation across EDs after adjustment for an array of standard socio-demographic and area-based variables. The context should be noted in that Northern Ireland has experienced a period of conflict, referred to as ‘the Troubles’ and that some geographical areas, and therefore hospital catchment areas were more affected by this conflict than others which may potentially impact on suicide risk of the people in those areas. There were some differences in the types of SH presenting to EDs, most notably the high proportion of people presenting at Hospital B with attempted drowning though this can be discounted as a significant explanation as the differences between hospitals persist even after adjusting for method and after excluding this group. Differences in the clinical severity of the self-harm act, however, are not specifically captured by the registry data, though this would be accounted for, to some extent, by the subsequent ED management and admission practices, which were measured. Very severe self-harm presentations may be brought to larger tertiary hospitals and, therefore, it is possible that these hospitals may have a different case-mix which is not evident from the data currently captured by the NIRSH. Another possibility is that the differences are due to the sizable variance across EDs in the proportion of patients who left before being seen or who refused treatment or admission [18], but this is a relatively small proportion of the overall numbers and again, the differences between EDs remained even after excluding this group.

Finally, we must consider the effects of the variations in management and next care across sites. The size of the variation in likelihood of admission following presentation with self-harm was large but in keeping with those previously documented elsewhere [16, 17], and such variation has been reported as reflecting resourcing and hospital practice rather than the needs of the presenting patients [16, 21]. We observed a large variation between presenting hospital in the proportion being admitted to a psychiatric hospital. This requires further analysis. It may reflect variation in models of provision of specialist psychiatric care in terms of home treatment vs. in-patient care provision offered in different areas. Another possibility is that direct admission to a psychiatric hospital from ED may be more likely if the psychiatric hospital is located close to the ED, as otherwise patients may be recorded as being admitted to the presenting general hospital initially. In practice, the association of different modalities of hospital care and patient management with subsequent suicide mortality will reflect a combination of confounding-by-indication (in which case worse outcomes, such as increased suicide risk for patients admitted to general or to psychiatric wards, can be expected), and the effects of effective and beneficial care (such as mental health assessment and subsequent indicated care) which might be associated with a lower suicide risk [26, 27]. Furthermore, many of the studies to date have lacked the power to examine suicide as an outcome relying on intermediate measures such as repeat presentation with self-harm or depression [28, 29]. However, most studies conclude that there is little clear evidence that routine aspects of SH care and patient management in ED, including psychosocial assessment, affect the risk of repeat SH or suicide, though acknowledge that it is difficult to be definitive because of heterogeneity amongst the SH population, variation in the types and quality of care received, and the methodological challenges of evaluating treatment effects using observational studies [5, 27, 30]. This paucity of good evidence about the effectiveness of intervention on short or longer-term outcomes is reflected in the recent NICE guidance [12].

Previous studies have indicated that the risk of suicide peaks in the months immediately following presentation with self-harm [8]. However, the absence, in the current study, of a significant impact of patient management at ED suggests, therefore, that the ED management and care per se, though important, is generally insufficient alone to change the trajectories of people presenting with SH. This implies that what happens, or does not happen, to the patients returning to the community in the period following an ED presentation is more important and that the connection between hospital and subsequent care by community based services is a critical element.

The gateway to accessing follow-up care after an ED attendance with self-harm is usually based on recommendations or referrals made by the mental health practitioner who assessed the person at the time of the ED attendance. Hence, this highlights the importance of ED staff referring all patients presenting with self-harm for a specialist mental health assessment as recommended by NICE guidance. It also highlights the importance of seamless pathways into follow-up mental health care regardless of whether that care is provided by statutory mental health services or other providers and removing barriers to engagement with such services, including stigma. In the absence of a diagnosable mental health problem, patients who self-harm may not be offered follow-up care due to the limited availability of services for this patient group. The evidence base for psychological intervention following self-harm has been growing in recent years and hence services for this group are becoming more readily available in keeping with the growing evidence. Even if patients are referred for specialist mental health assessment and follow-up care is recommended, it is recognized that patients may have long waits for community-based appointments and may have difficulty engaging with the follow-up care offered, and that such patients may be at particular risk of suicide [29]. It is likely therefore, that the hospitals and EDs identifiers in the current study are proxy markers for the availability, capacity, quality, timeliness of access to and engagement with follow-up psychiatric or other mental health support services in the wider community in which the EDs are situated. It is, therefore, important that such services are available to meet the needs of this population and that services are commissioned on the basis of need rather than hospital admission rates which are known to vary [16].

The study has some notable strengths, including the use of an established self-harm register with population-wide coverage and quality assured data extraction processes and linkage to official mortality records for the entire cohort. This facilitated excellent coverage of risk factors known to be associated with suicide risk following SH and the care and management received at each ED. On the other hand, the data are limited to that recorded by clinical staff at time of ED presentation, and some variation in the clinical recording across sites cannot be entirely ruled. It is also recognised that there is difficultly in making assumptions that all those who are ‘referred’ for psychosocial assessment actually have this carried out. Conversely, the NIRSH may under-estimate referrals for mental health assessment as it does not capture referrals for assessments that are made after patients have been admitted to a ward that were not recorded in the ED notes. We have also classified patients according to the ED of first attendance, though analysis within the Registry indicates that most patients attend their local ED. We acknowledge that only broad categories of ED management are recorded and not the intensity, duration, or quality of care provided during the hospital attendance. These are recognised limitations of administrative data. Finally, as this study covers an eight-year period that has seen some dramatic changes in the organisation and provision of care and management of patients attending EDs with self-harm, it could be argued that the mortality differences for the period studied do not reflect current patterns. However, an analysis restricted to the second half of the study period shows a very similar pattern.

In conclusion, this study has demonstrated a marked variation in suicide risk for patients attending different EDs with SH. Although mortality risk was associated with referral for psychosocial assessment and subsequent type of care patients received in terms of admission to hospital. These differences may be attributable to what happens after the ED attendance in terms of services offered and the degree of engagement with follow-up assessment and other services. The well-attested and marked elevated risk of suicide (and deaths from external causes) in the months immediately following ED attendance underlines the importance of care in this critical period both at the time of the ED attendance and the months thereafter. It is important to maximise the proportion of people receiving a specialist psychosocial assessment at the time of ED attendance as our study has confirmed lower mortality for those who have an assessment while in the ED. If this is not carried out while in the ED it can be very difficult to maintain contact with this group once this opportunity has passed and they leave the ED. The reasons for these difficulties are likely to be manifold. It is, therefore, recommended that there is an enhanced focus on ensuring initial assessment before leaving the ED/ward and maximising engagement with follow-up services, if required. Given the risk of death from both suicide and other external causes there should be more emphasis on the quality and timing of immediate post-discharge care and the links between the ED, community mental health services, primary care settings and other relevant support services for mental health and related issues such as drug and alcohol misuse. It is important that such services are available to meet the needs of this population and that services are commissioned on the basis of need and do not relay on self-harm hospital admission rates which are known to vary [16], and bearing in mind that only a minority of self-harm cases present to the ED. Finally, and in keeping with the NICE recommendations [12], we do not advocate risk identification based on variations in mortality risk according to ED attended, though these data do suggest that there is perhaps a greater need to examine the continuity and uptake of services in some localities.

## Data Availability

The data used in this study are available in the Honest Broker Service (HBS) within the Business Services Organisation Northern Ireland (BSO), but as restrictions apply, they are not publicly available. All proposals to use data are subject to review by an independent HBS Governance Board (HBSGB). Before any data can be accessed, approval must be given by the HBSGB. When access has been granted, it is gained through a privacy protecting safe haven and remote access.
